# Supplementary material for: Comparison of SARS-CoV-2 Evolution in Paediatric Primary Airway Epithelial Cell Cultures Compared with Vero-Derived Cell Lines
Source: Viruses. 2022 Feb 5;14(2):325. doi: 10.3390/v14020325 (PMC8877208; doi:10.3390/v14020325)
Supplement: Supplementary file 1 [file viruses-14-00325-s001.zip › viruses-1521794-supplementary.pdf]

PHE VEROE6

POS	REFERENCE	VARIANT	P1	P2	P3	P4A	P4B	CATEGORY	GENE	S/NS?	CONSEQUENCE	PROTEIN
7749	C	T	0.1	12	21	16	19	MINOR	ORF1AB	NS	T2495I	NSP3
8782	C	T	97	98	97	97	98	CORE	ORF1AB	S		
9534	C	T	0.1	0.1	2	5	5	MINOR	ORF1AB	NS	T3090I	NSP4
18488	T	C	98	97	97	98	96	CORE	ORF1AB	NS	I6075T	NSP14
19983	C	CT	7	6	7	9	8	MINOR	ORF1AB	NS	FRAMESHIFT. D6576*	NSP15
21697	C	T	13	8	4	24	15	MINOR	ORF1AB	S		
21761	GCTATACATGTCTCTGGGACCAATGGTA	G	0.1	0.1	2	10	6	MINOR	SPIKE	NS	IHVSGTNGT67-76del	SPIKE
21846	C	T	17	47	3	0.1	0.1	MINOR	SPIKE	NS	T95I	SPIKE
22100	G	A	0.1	0.1	0.1	11	15	MINOR	SPIKE	NS	E180K	SPIKE
22206	A	G	0.1	0.1	0.1	8	11	MINOR	SPIKE	NS	D215G	SPIKE
**23605**	**T**	**G**	**97**	**94**	**0.1**	**0.1**	**0.1**	**VARIANT**	**SPIKE**	**S**		
**23597**	**AATTCTCCTCGGCGGGCACGTAGTG**	**A**	**0.6**	**4**	**89**	**94**	**94**	**VARIANT**	**SPIKE**	**NS**	**NSPRRARSV679I**	**SPIKE**
25339	C	T	0.1	7	0.1	0.1	0.1	MINOR	SPIKE	S		SPIKE
26353	C	T	0.1	0.1	7	16	19	MINOR	E	NS	L37F	E
26354	T	G	0.1	0.1	0.1	5	5	MINOR	E	NS	L37R	E
28144	T	C	96	98	98	97	97	CORE	ORF8	NS	L84S	ORF8
28833	C	T	0.1	22	0.1	0.1	0.1	MINOR	N	NS	S187L	N
29366	C	T	7	34	2	0.1	0.1	MINOR	N	NS	P365S	N
29596	A	G	98	98	98	98	98	CORE	ORF10	NS	I13M	ORF10
29637	T	C	0.1	0.1	0.1	6	5	MINOR	ORF10	NS	I27T	ORF10
29844	AT	A	0.1	11	0.1	0.1	0.1	MINOR	3' UTR	S		NC

PHE WD-PNECS

POS	REFERENCE	VARIANT	P1	P2	P3	P4		CATEGORY	GENE	S/NS?	CONSEQUENCE	PROTEIN
241	C	T	0.1	0.1	6	0.1		MINOR	5'UTR	S		
514	TGTTATG	T	2	7	0.1	0.1		MINOR	ORF1AB	NS	MV85DEL	NSP1
1593	C	T	0.1	0.1	0.1	35		MINOR	ORF1AB	NS	S443F	NSP2
2994	A	C	0.1	0.1	0.1	7		MINOR	ORF1AB	NS	E910A	NSP3
4455	C	T	0.1	15	0.1	0.1		MINOR	ORF1AB	NS	A1397V	NSP3
4928	A	T	0.1	0.1	0.1	46		MINOR	ORF1AB	NS	N1555Y	NSP3
6696	C	CT	3	8	0.1	1		MINOR	ORF1AB	NS	L2146S*	NSP3
6750	A	G	0.1	7	0.1	0.1		MINOR	ORF1AB	NS	N2162S	NSP3
7444	A	G	0.1	0.1	10	0.1		MINOR	ORF1AB	S		
7749	C	T	0.1	7	0.1	0.1		MINOR	ORF1AB	NS	T2495I	NSP3
7866	G	T	0.1	0.1	6	0.1		MINOR	ORF1AB	NS	G2534V	NSP3
8782	C	T	97	99	99	99		CORE	ORF1AB	S		
9532	C	T	0.1	15	0.1	0.1		MINOR	ORF1AB	S		
11074	C	CT	1	5	0.1	0.1		MINOR	ORF1AB	NS	L3606F*	NSP6
12809	C	T	0.1	0.1	0.1	5		MINOR	ORF1AB	NS	L4182F	NSP9
12860	A	G	0.1	0.1	10	0.1		MINOR	ORF1AB	NS	S4199G	NSP9
13604	G	A	0.1	0.1	7	0.1		MINOR	ORF1AB	NS	R4447H	NSP12
16949	C	T	0.1	0.1	13	0.1		MINOR	ORF1AB	NS	P5562L	NSP13
17440	C	T	0.1	35	0.1	0.1		MINOR	ORF1AB	NS	P5726S	NSP13
18063	TA	T	0.1	0.1	9	0.1		MINOR	ORF1AB	NS	frameshift	
18488	T	C	98	97	96	97		CORE	ORF1AB	NS	I6075T	NSP14
18508	C	T	0.1	0.1	0.1	8		MINOR	ORF1AB	NS	L6082F	NSP14
19983	C	CT	7	7	0.1	6		MINOR	ORF1AB	NS	FRAMESHIFT. D6576*	NSP15
20178	C	T	0.1	0.1	0.1	5		MINOR	ORF1AB	S		
21697	C	T	13	0.1	8	0.1		MINOR	SPIKE	S		
21846	C	T	17	0.1	18	2		MINOR	SPIKE	NS	T95I	Spike
23277	C	T	0.1	0.1	10	0.1		MINOR	SPIKE	NS	T572I	Spike
23582	T	C	0.1	0.1	9	0.1		MINOR	SPIKE	NS	Y674H	Spike
**23597**	**AATTCTCCTCGGCGGGCACGTAGTG**	**A**	**6**	**11**	**0**	**2**		**MINOR**	**SPIKE**	**NS**	**NSPRRARSV679I**	**Spike**
**23605**	**T**	**G**	**97**	**86**	**94**	**95**		**CORE**	SPIKE	**S**		
26681	C	T	0.1	0.1	7	0.1		MINOR	M	S		M
27434	C	T	0.1	0.1	6	0.1		MINOR	ORF7A	NS	T14I	7A
27509	C	T	0.1	0.1	6	0.1		MINOR	ORF7A	NS	T39I	7A
27814	TTG	T	0.1	0.1	10	0.1		MINOR	ORF7B	NS	FRAMESHIFT	7B
28144	T	C	96	97	96	98		CORE	ORF8	NS	L84S	8
28393	T	C	0.1	11	0.1	0.1		MINOR	ORF8	S		
29274	C	T	0.1	23	0.1	0.1		MINOR	N	NS	T334I	N
29366	C	T	7	0.1	17	0.1		MINOR	ORF10	NS	P365S	N
29596	A	G	98	98	98	99		CORE	10	NS	I13M	10

BT20.1 VERO

POS	REFERENCE	VARIANT	P1	P2	P3	P4		CATEGORY	GENE	S/NS?	CONSEQUENCE	PROTEIN
241	C	T		100	100	100		CORE	5' UTR	S		
635	C	T		7	0.1	0.1		MINOR	ORF1AB	NS	R124C	NSP1
1420	C	T		98	99	99		CORE	ORF1AB	S		
1681	G	A		99	99	99		CORE	ORF1AB	S		
3037	C	T		98	99	99		CORE	ORF1AB	S		
6255	C	T		96	99	96		CORE	ORF1AB	NS	A1997V	NSP3
**10870**	**G**	**T**		0.1	**90**	**96**		**VARIANT**	ORF1AB	**S**		
**14318**	**C**	**T**		0.1	**85**	**96**		**VARIANT**	ORF1AB	**NS**	**T4685I**	**NSP12**
14408	C	T		98	97	98		CORE	ORF1AB	NS	P4715L	NSP12
16293	CATACGT	C		0.1	0.1	7		MINOR	ORF1AB	NS	CHANGE	
19983	C	CT		8	6	7		MINOR	ORF1AB	NS	FRAMESHIFT. D6576*	NSP15
22311	C	T		6	0.1	0.1		MINOR	SPIKE	NS	T205I	SPIKE
23403	A	G		97	98	99		CORE	SPIKE	NS	D614G	SPIKE
**23997**	**C**	**G**		0.1	**84**	**96**		**VARIANT**	SPIKE	**NS**	**P812R**	SPIKE
25314	G	T		3	5	0.1		MINOR	SPIKE	NS	G1251V	SPIKE
25317	C	G		3	5	7		MINOR	SPIKE	NS	S1252C	SPIKE
25521	C	T		18	0.1	7		MINOR	ORF3A	S		
27208	C	T		8	0.1	0.1		MINOR	ORF6	NS	H3Y	ORF6
27213	C	T		9	2	0.1		MINOR	ORF6	S		
27671	TTCAAG	T		100	96	96		CORE	ORF7A	NS	FRAMESHIFT and truncation	ORF7A
**28253**	**C**	**T**		**19**	**93**	**99**		**VARIANT**	**ORF8**	**S**		
28881	G	A		97	98	99		CORE	N	NS	R203K	N
28882	G	A		96	99	99		CORE	N	S		
28883	G	C		99	99	100		CORE	N	NS	G204R	N

BT20.1 WD-PNECS

POS	REFERENCE	VARIANT	P1	P2	P3	P4		CATEGORY	GENE	S/NS?	CONSEQUENCE	PROTEIN
241	C	T		99	100	99		CORE	5' UTR	S	.	
1420	C	T		99	100	97		CORE	ORF1AB	S		
1681	G	A		97	98	99		CORE	ORF1AB	S		
3037	C	T		98	99	99		CORE	ORF1AB	S		
6255	C	T		91	89	93		CORE	ORF1AB	NS	A1997V	NSP3
**6683**	**AATT**	**A**		**46**	**45**	**52**		**VARIANT**	**ORF1AB**	**NS**		
14408	C	T		99	98	98		CORE	ORF1AB	NS	P4715L	NSP12
19983	C	CT		7	6	8		MINOR	ORF1AB	NS	FRAMESHIFT. D6576	NSP15
21101	G	GT		1	5	0.1		MINOR	ORF1AB	NS	FRAMESHIFT. F6948	NSP16
23403	A	G		97	99	98		CORE	SPIKE	NS	D614G	SPIKE
25314	G	T		0.1	6	1		MINOR	SPIKE	NS	G1251V	SPIKE
25317	C	G		0.1	6	1		MINOR	SPIKE	NS	S1252C	SPIKE
27671	TTCAAG	T		100	98	100		CORE	ORF7A	NS		
28881	G	A		99	97	99		CORE	N	NS	R203K	N
28882	G	A		96	97	99		CORE	N	S		
28883	G	C		99	99	99		CORE	N	NS	G204R	N
29418	T	A		0.1	5	0.1		MINOR	N	NS	L382*	N

Table S1. Frequency of variants in reference to Wuhan-Hu-1 identified in this study. Variants only shown where there was at least one instance of frequency >5%. Where undetectable an arbitrary value of 0.1 was assigned. Frequency data is highlighted by colour (green for higher, yellow for lower). Mutations have been assigned status of core, variant or mi-

nor. "Variant" mutations, which are shown in the main figures, have been highlighted in bold text. Additionally, for each variant, data for nucleotide location, reference and variant nucleotides, gene & protein location, and consequence (e.g., synonymous [S] or non-synonymous [NS]) are shown.

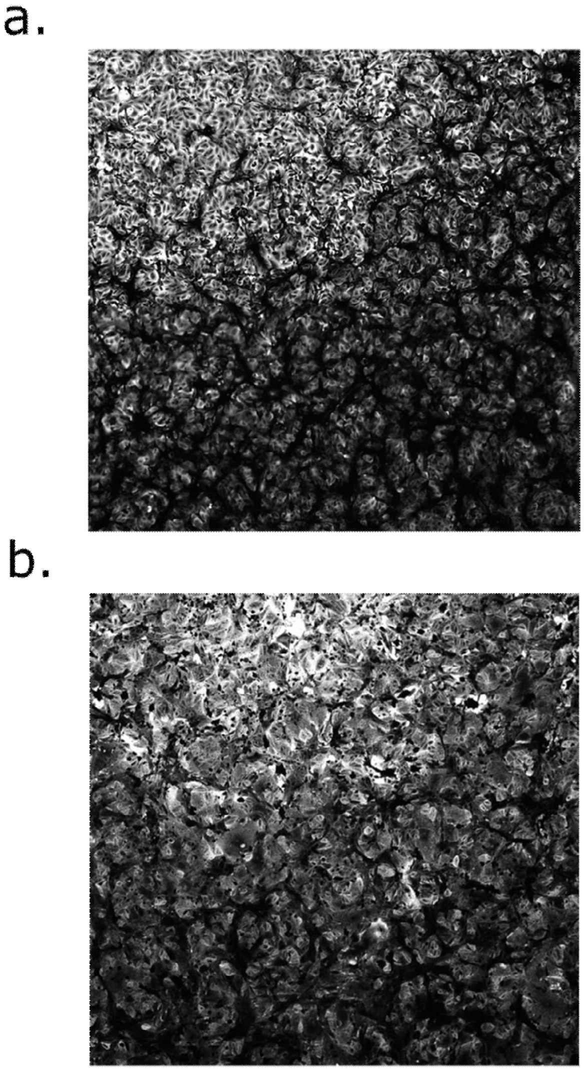

Figure S1. Fusogenicity of PHE and BT20.1 P4 on Vero cells. Higher magnification images of plaque visualisation of PHE (a) and BT20.1 (b) P4 on Vero cells from the same images shown in Figure 1.

a.

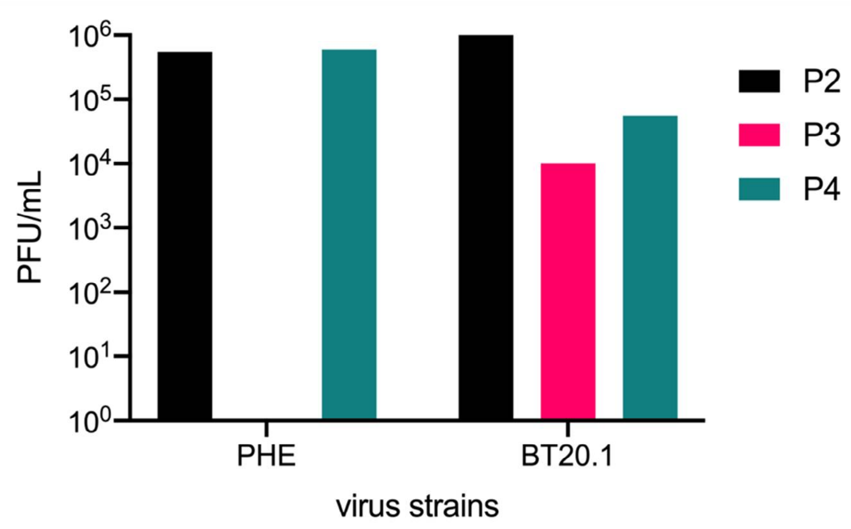

Figure S2. Growth kinetics of PHE and BT20.1 during passage in WD-PNECs. Infectivity titres for material generated from isolation/passage of PHE and BT20.1 on WD-PNECs.

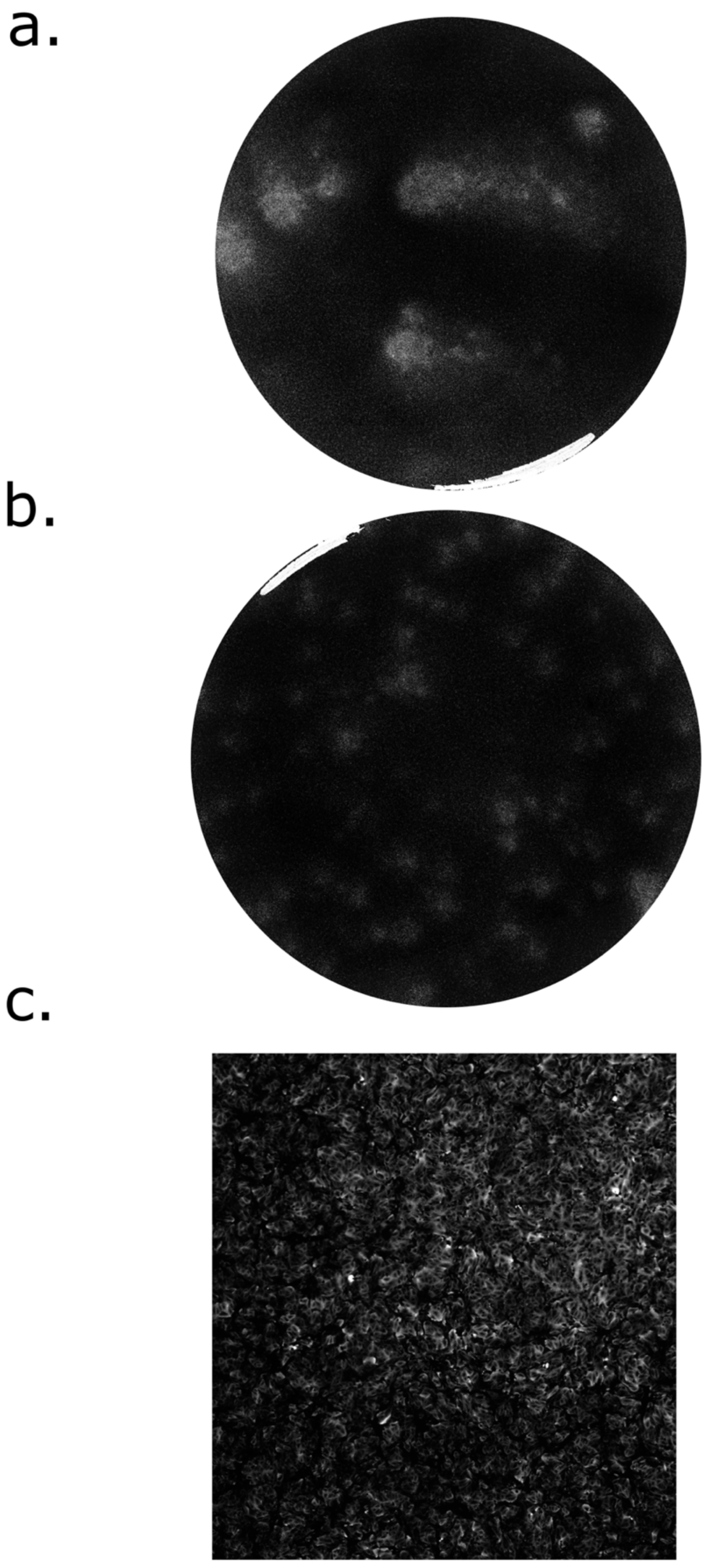

Figure S3. Plaque morphology of SARS-CoV-2 grown in WD-PNECs. Plaque visualisation of PHE (a) and BT20.1 (b) P4 on Vero cells. Higher magnification images of plaque visualisation of BT20.1 (c) P4 on Vero cells from the same images shown (b).

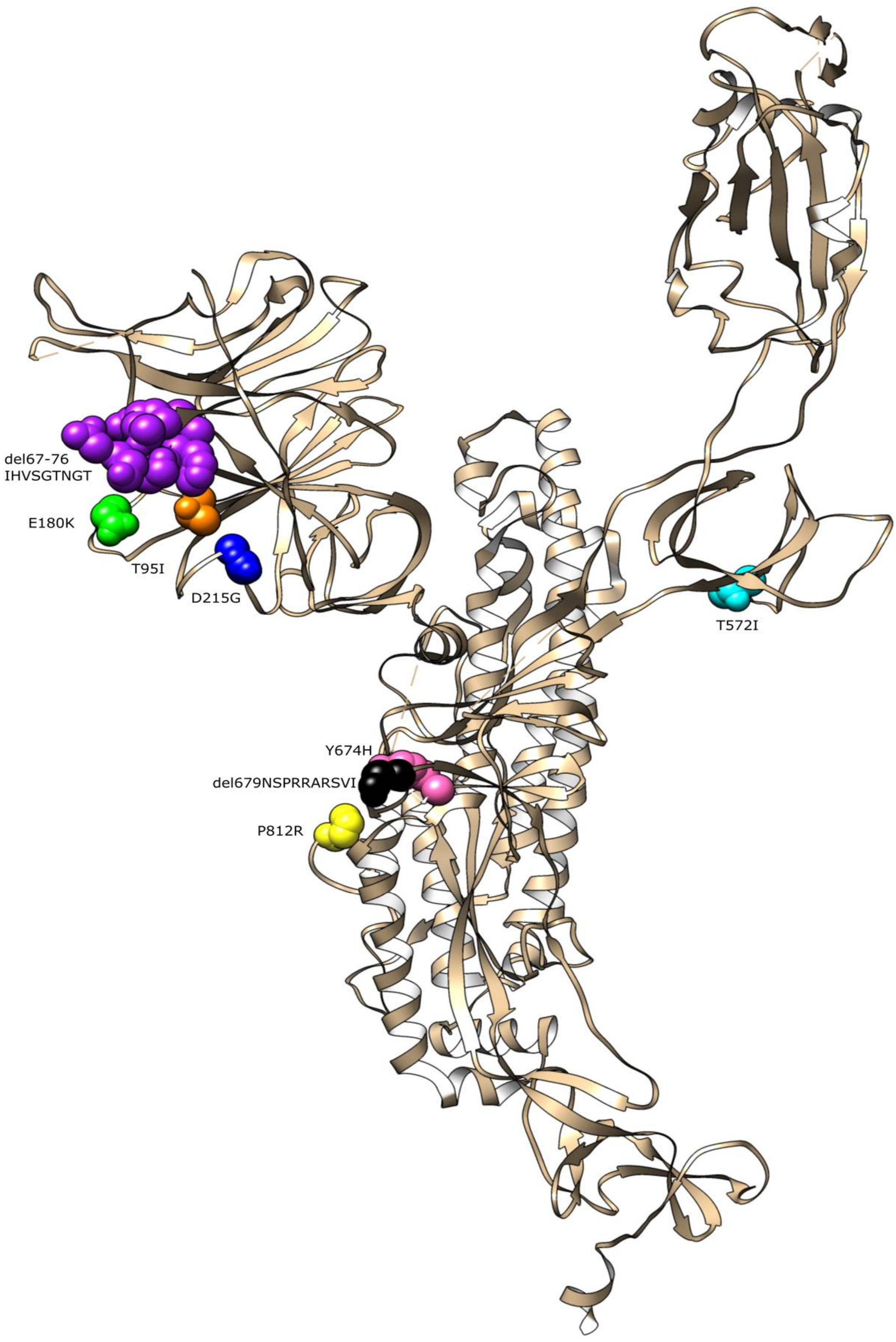

Figure S4. Location of Spike mutant variants observed in this study on model structure of a single Spike monomer in the pre-fusion state (PDB 7C2L from [40]). Variants identified in the Spike cytoplasmic tail (G1251V and S1252C) are not shown.
